# *Microbial Biotechnology* at the Frontiers of Innovation: Engineering Solutions for Health, Industry and Sustainability

**DOI:** 10.1111/1751-7915.70440

**Published:** 2026-09-07

**Authors:** Patricia Bernal, Juan Luis Ramos

**Affiliations:** ^1^ Departmento de Microbiología, Facultad de Biología Universidad de Sevilla Sevilla Spain; ^2^ Estación Experimental del Zaidín Consejo Superior de Investigaciones Científicas Granada Spain

The most visited posts on the *Microbial Biotechnology* journal's social networks during the second trimester of 2026 illustrate the remarkable diversity of contemporary research in this field. Microorganisms are no longer investigated solely to understand their biology or exploit their biotechnological properties; they are increasingly engineered as versatile platforms to address challenges in medicine, agriculture, industrial manufacturing, and environmental sustainability. *Microbial Biotechnology* followers are interested in advances in molecular microbiology, synthetic biology, systems biology, protein engineering and bioprocess engineering, and how these fields converge to transform fundamental microbial discoveries into practical applications. This editorial illustrates how the choices of *Microbial Biotechnology* followers exemplify the emerging trends that are shaping the future of biotechnology.

## 1 Microbial Pathogenesis and Antimicrobial Innovation

One important topic is the development of innovative antimicrobial strategies that target microbial virulence rather than survival (Abbas et al. 2024). Wang et al. described in *Microbial Biotechnology* (https://doi.org/10.1111/1751‐7915.70321) the identification of alcohol dehydrogenase 1 (Adh1) as a previously unrecognized regulator of *Candida albicans* morphogenesis through modulation of SNF1 phosphorylation. The authors demonstrated that 2‐hydroxyanthraquinone inhibits hyphal formation and biofilm development by targeting this pathway, providing a promising anti‐virulence approach that may reduce the selective pressure associated with conventional antifungal therapies. This work aligns with current trends in addressing fungal signalling networks to develop next‐generation therapeutics aimed at controlling pathogenicity instead of simply eliminating pathogens.

## 2 Engineering Microbial Cell Factories

Conventional and non‐conventional cell factories are attracting continuous interest as efficient production platforms for high‐value biomolecules (Nandan and Datta 2026). Yan et al. in *Microbial Biotechnology* (https://doi.org/10.1111/1751‐7915.70318) showed that *Trichoderma reesei*, traditionally exploited for industrial enzyme production, can be successfully engineered for extracellular production of insulin glargine. By optimizing secretion signals, fusion partners, and endoplasmic reticulum stress responses, the authors substantially improved recombinant protein secretion, thereby expanding the potential of filamentous fungi as hosts for therapeutic protein manufacturing. Another study by Li et al. in *Microbial Biotechnology* (https://doi.org/10.1111/1751‐7915.70338) redesigned membrane trafficking pathways in *Saccharomyces cerevisiae* to enhance extracellular vesicle production. This process introduces new generic approaches for the manufacture of extracellular vesicles that may serve as future therapeutic delivery systems. These studies are in line with current trends that advance host engineering to transform them into increasingly sophisticated and versatile cell factories (Calero and Nikel 2019).

## 3 Engineering Enzymes for Sustainable Biocatalysis

Protein engineering continues to expand the catalytic capabilities of microbial enzymes (Miller et al. 2022). Fucosylated oligosaccharides, with applications in infant nutrition and glycobiology, are of interest in the food industry. Vodičková et al., in *Microbial Biotechnology* (https://doi.org/10.1111/1751‐7915.70354), converted an α‐L‐fucosidase from *Paenibacillus thiaminolyticus* into an efficient transfucosidase through rational mutagenesis, shifting catalytic activity from hydrolysis towards transglycosylation. The engineered enzyme enables the selective synthesis of valuable chemicals and has potential for the development of biosensors.

Likewise, Kato et al., in *Microbial Biotechnology* (https://doi.org/10.1111/1751‐7915.70369), engineered the previously uncharacterized cytochrome P450 CYP107J1 from *Bacillus subtilis* into a hydrogen peroxide‐driven peroxygenase using only two amino acid substitutions. The redesigned enzyme displayed dramatically improved catalytic activity and expanded substrate specificity, including efficient indigo synthesis, demonstrating how minimal structural modifications can unlock entirely new biocatalytic functions. Advances in genetic engineering of proteins are also in line with discovery programmes aimed at identifying industrial enzymes from metagenomic libraries and screening programmes (Ferrer et al. 2016; Lorenz and Eck 2005). These studies exemplify the growing ability to redesign microbial enzymes for sustainable chemical synthesis.

## 4 Intelligent Bioprocess Engineering

Engineering increasingly extends beyond microbial genomes to encompass the design of adaptive manufacturing processes. The contribution by Delvenne et al. in *Microbial Biotechnology* (https://doi.org/10.1111/1751‐7915.70329) introduces ‘Segregostat’, a real‐time cell–machine interface that combines automated flow cytometry with dynamic process control to maintain stable microbial physiological states under industrially relevant perturbations. By continuously monitoring and adjusting microbial populations, this platform represents an important step towards intelligent, automated bioprocessing capable of improving scalability and manufacturing robustness. The integration of systems biology with digital process control reflects a broader movement towards smart biomanufacturing.

## 5 *Microbial Biotechnology* for Agriculture

Plant growth promoting microorganisms and organisms with biocontrol properties are at the core of the development of suitable practices to replace chemical fertilizers. In this line, *Microbial Biotechnology* is contributing innovative solutions for sustainable agriculture (Singh et al. 2025). Grace et al. in *Microbial Biotechnology* (https://doi.org/10.1111/1751‐7915.70394) reported the first bacteriophages isolated to act against the bacterial pathogens associated with acute oak decline, in particular *Brennera goodwinii* and *Gibbsiella quercinencis*. Although the phages effectively inhibited bacterial growth under laboratory conditions, their limited performance in oak saplings underscores the ecological complexity of translating phage therapy into field applications. Rather than representing a limitation, these findings highlight the importance of integrating ecological understanding with microbiological innovation when developing biological control strategies for crop and forest protection. Biocontrol by phages is a trend‐recovering practice established in the last century and forgotten due to advances in antibiotic development; however, with the current antimicrobial resistance crisis, phages can become the stars.

## 6 Vaccine Engineering and Nanobiotechnology

COVID‐19 has awakened the vaccine field with highly surprising advances in mRNA vaccines and fast and reliable vaccines against viral, fungal and bacterial infections (Brüssow 2023). Vaccination is paramount to prevent disease spread, and advances in recombinant technologies continue to reshape vaccine development.

Byun et al. in *Microbial Biotechnology* (https://doi.org/10.1111/1751‐7915.70391) described a recombinant nanoparticle vaccine against West Nile virus based on the cholera toxin B subunit displaying the viral envelope domain III antigen. The engineered nanoparticles elicited strong neutralizing antibody responses while minimizing cross‐reactivity with related flaviviruses, supporting their potential as safe and scalable vaccine platforms for emerging infectious diseases. This work illustrates how nanobiotechnology and recombinant protein engineering are converging to create next‐generation vaccines with improved efficacy and specificity.

## 7 *Microbial Biotechnology* for Environmental Sustainability

Environmental sustainability remains one of the defining challenges driving *Microbial Biotechnology*. Phale et al., in *Microbial Biotechnology* (https://doi.org/10.1111/1751‐7915.70372), presented a systems‐level perspective on bacterial biodegradation of aromatic pollutants, arguing that successful bioremediation depends not only on metabolic pathways but also on coordinated traits such as environmental sensing, chemotaxis, stress tolerance, substrate transport, regulatory networks, and microbial cooperation. Their ‘arsenal’ concept reframes biodegradation as an emergent property of complex microbial systems.

A complementary example is provided by the study on PET upcycling by Molpeceres‐García et al., in *Microbial Biotechnology* (https://doi.org/10.1111/1751‐7915.70340), which integrates chemical depolymerization with synthetic biology to transform plastic waste into biodegradable polyhydroxyalkanoates (PHAs). PET was rapidly converted into BHET by glycolysis, after which an engineered consortium of *Comamonas testosteroni* and *Pseudomonas putida* cooperatively metabolized PET‐derived intermediates into valuable bioplastics. By combining chemical recycling with microbial metabolism, this work demonstrates a promising strategy for developing a circular plastics economy.

These studies illustrate how *Microbial Biotechnology* is increasingly positioned at the intersection of environmental remediation, synthetic biology and industrial biotechnology.

## 8 Looking Ahead

Collectively, these contributions reveal a field undergoing a profound transformation. Modern *Microbial Biotechnology* is increasingly centred on redesigning microbial systems to solve practical problems. Whether engineering enzymes for sustainable catalysis, constructing microbial cell factories for therapeutic production, developing intelligent bioprocesses, designing recombinant vaccines, controlling plant diseases through bacteriophages, or converting plastic waste into biodegradable materials, the common thread is the integration of mechanistic biological knowledge with engineering principles. Therefore, *Microbial Biotechnology* is evolving into a truly engineering‐driven science, one capable of delivering scalable solutions for healthcare, agriculture, industrial manufacturing and environmental sustainability.

## Author Contributions

**Patricia Bernal:** conceptualization, writing – original draft, writing – review and editing. **Juan Luis Ramos:** conceptualization, writing – original draft, writing – review and editing.

## Funding

The authors have nothing to report.

## Conflicts of Interest

The authors declare no conflicts of interest.

## Data Availability

Data sharing not applicable to this article as no datasets were generated or analysed during the current study.
